# Assessing spinal movement during four extrication methods: a biomechanical study using healthy volunteers

**DOI:** 10.1186/s13049-022-00996-5

**Published:** 2022-01-15

**Authors:** Tim Nutbeam, Rob Fenwick, Barbara May, Willem Stassen, Jason E. Smith, Jono Bowdler, Lee Wallis, James Shippen

**Affiliations:** 1 Emergency Department, University Hospitals Plymouth NHS Trust, Plymouth, UK; 2 Devon Air Ambulance Trust, Exeter, UK; 3 Division of Emergency Medicine, University of Cape Town, Cape Town, South Africa; 4 University Hospitals Birmingham, Birmingham, UK; 5 Institute for Future Transport and Cities, University of Coventry, Coventry, UK; 6 Academic Department of Military Emergency Medicine, Royal Centre for Defence Medicine, Birmingham, UK; 7 Fire and Rescue Service Trainer, Severn Park Fire and Rescue Centre, Bristol, UK

## Abstract

**Background:**

Motor vehicle collisions are a common cause of death and serious injury. Many casualties will remain in their vehicle following a collision. Trapped patients have more injuries and are more likely to die than their untrapped counterparts. Current extrication methods are time consuming and have a focus on movement minimisation and mitigation. The optimal extrication strategy and the effect this extrication method has on spinal movement is unknown. The aim of this study was to evaluate the movement at the cervical and lumbar spine for four commonly utilised extrication techniques.

**Methods:**

Biomechanical data was collected using inertial Measurement Units on 6 healthy volunteers. The extrication types examined were: roof removal, b-post rip, rapid removal and self-extrication. Measurements were recorded at the cervical and lumbar spine, and in the anteroposterior (AP) and lateral (LAT) planes. Total movement (travel), maximal movement, mean, standard deviation and confidence intervals are reported for each extrication type.

**Results:**

Data from a total of 230 extrications were collected for analysis. The smallest maximal and total movement (travel) were seen when the volunteer self-extricated (AP max = 2.6 mm, travel 4.9 mm). The largest maximal movement and travel were seen in rapid extrication extricated (AP max = 6.21 mm, travel 20.51 mm).

The differences between self-extrication and all other methods were significant (*p* < 0.001), small non-significant differences existed between roof removal, b-post rip and rapid removal.

Self-extrication was significantly quicker than the other extrication methods (mean 6.4 s).

**Conclusions:**

In healthy volunteers, self-extrication is associated with the smallest spinal movement and the fastest time to complete extrication. Rapid, B-post rip and roof off extrication types are all associated with similar movements and time to extrication in prepared vehicles.

## Background

Motor vehicle collisions (MVC’s) are a common cause of serious injury and death—accounting for 1.3 million deaths and 50 million serious injuries per annum worldwide [1]. Up to 40% of casualties injured following an MVC will remain trapped—these casualties are more likely to die than their un-trapped counterparts [2–8].

Casualties who remain in their vehicle following an MVC will belong in one of four groups: (i) The casualty can self-extricate or extricate with minimal assistance (self-extrication), (ii) the casualty is unable to self-extricate due to pain, their psychological response to the incident or their injuries but can be assisted from the vehicle (assisted extrication) (iii) the casualty is either advised or chooses not to self-extricate due to concern of exacerbating injury (particularly spinal injury) by movement (medically trapped), (iv) the casualty is physically trapped in the vehicle (e.g. due to displaced road furniture) or requires disentanglement from the vehicle wreckage by rescue services (disentanglement and rescue) [9]. These groups are not mutually exclusive and a patient may belong in more than one group across their extrication experience.

The role of the rescue services will be different for each casualty group. For example, casualties who can self-extricate will require minimal or no intervention from rescue services but those needing disentanglement and rescue will require the use of cutting and spreading tools [10]. Casualties in the assisted extrication (assisted) and medically trapped (medical) groups can be encouraged to self-extricate, have a rapid extrication (without the use of tools, sometimes referred to as a B plan) or can alternatively have a more traditional extrication, where the vehicle is cut away from around the casualty to improve access and offer an alternative route of egress (sometimes referred to as an A plan extrication) [10].

The approach of the rescue service is based on movement minimisation and mitigation, primarily to avoid exacerbating a primary spinal injury [11].The role of small movements in this is unknown and a challenge to accurately quantify. Large or forceful movements are considered higher risk than smaller movements^1^. Rescue service teaching recommends that casualties in the assisted or medical groups receive a traditional extrication method, as it is understood that these result in less spinal movement than other techniques [11]. Recently these principles have been challenged; with a number of small biomechanical studies demonstrating that self-extrication may cause less movement than more traditional extrication techniques [12–14].

Self-extrication or rapid techniques may be superior to traditional A plan techniques in relation to casualty and operational factors. Firstly the use of extrication tools is not a benign intervention and may cause considerable and costly vehicular damage, will have significant resource implications (both human and equipment), is physically demanding and may also subject casualties and rescuers to a real risk of harm [15]. Secondly, traditional extrication techniques can take a significant amount of time, with a median time of 30 min across traditional extrication types [16]. Whilst a patient remains entrapped the ability of clinicians to provide meaningful patient assessment and intervention is limited [17]. The extended time frame associated with traditional extrication and the delays this causes in accessing care may be factors that contribute to the excess mortality and morbidity seen in trapped patients [8]

We have previously demonstrated that spinal cord injuries occur in 0.7% of patients trapped following an MVC [8]. However, before any change in practice can be recommended, a detailed understanding of the movement of the spine associated with each of the commonly used extrication techniques to support a rigorous comparison of such techniques is important. This study will assess the three most commonly performed extrication techniques along with self-extrication and the resulting spinal movement (Box 1) [18].

**BOX 1:** Extrication procedures assessed and method of assessment

**Fig. 1:**
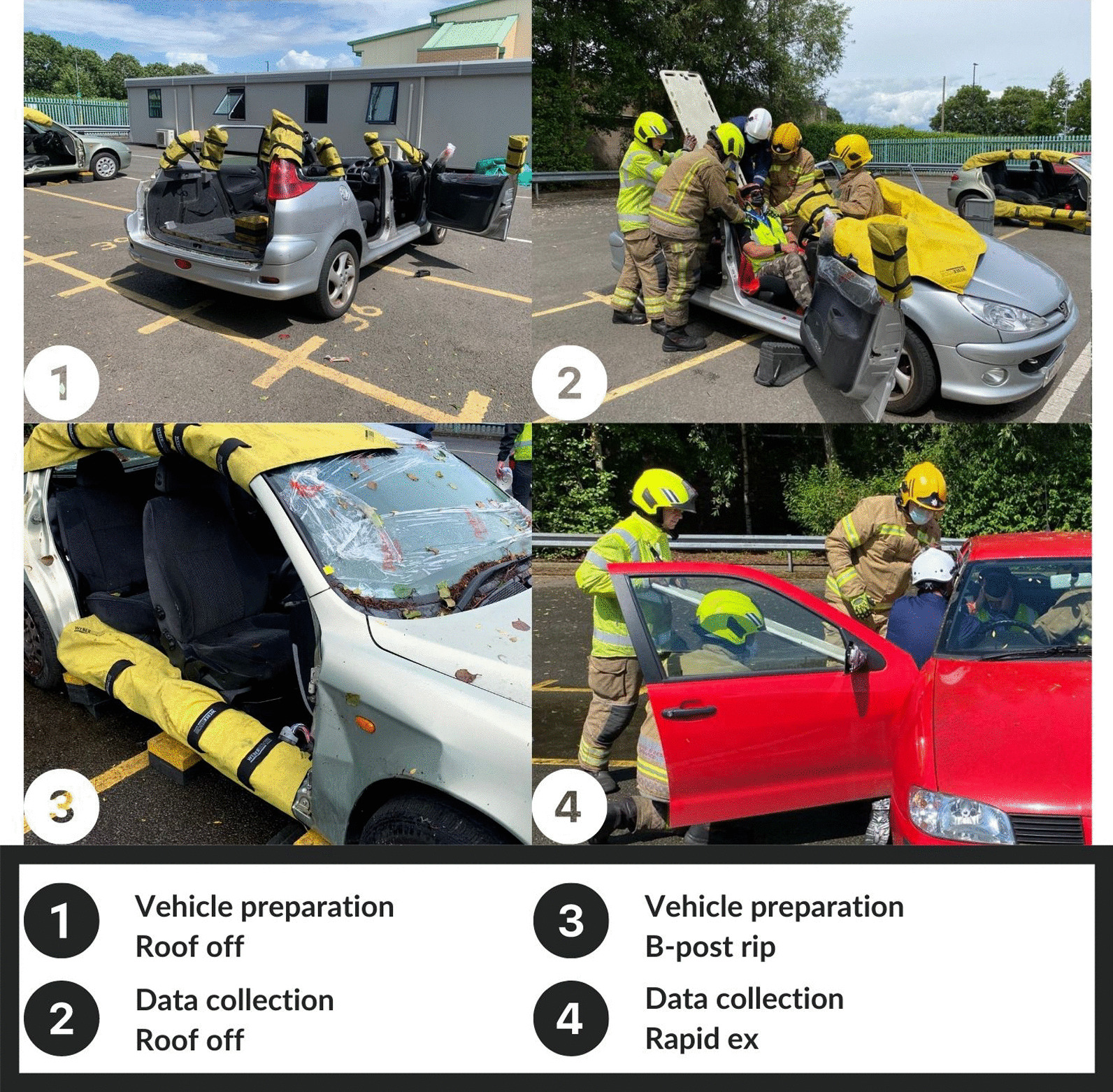
Vehicle preparation and data collection

**Roof removal:** The A, B and C posts and the roof removed facilitating a vertical extrication technique (Fig. 1) Study car preparation: the vehicle was stabilised, all posts were cut, the roof was removed and sharp edges were made safe Study vehicle: Peugeot 307 5 door, 2004 Technique: The participant was provided with Manual In-Line Neck Stabilisation (MILNS) throughout, the back support of the driver’s seat was reclined mechanically and the Long Spinal Board (LSB) inserted to the seat base. The participant was then slid up the board until they were horizontally situated (securely) on the LSB **B-post rip:** The B-post, drivers and drivers side rear door are removed to facilitate patient access and horizontal extrication (Fig. 1) Study car preparation: The vehicle was stabilised, B-post was removed completely using two cuts and all sharps were made safe Study vehicle: Peugeot 307 5 door, 2006 Technique: The participant was provided with MILNS throughout. The back support of the driver’s seat was reclined mechanically. The LSB was inserted at an oblique angle (pointed towards front centre console) and inserted to the seat base. Participant was then slid up the LSB until fully on the board at which point the LSB is rotated 45 degrees and placed horizontally onto the floor, next to the vehicle **Rapid:** The driver’s door is opened and the casualty assisted with a lateral extrication technique Study car preparation: The driver’s door was opened and the maximal opening angle enhanced using firefighter body weight only Study vehicle: Seat Ibiza 5 door, 1999 Technique: The drivers door is opened. The participant was provided with MILNS throughout. The LSB was inserted under the right thigh and hip, through an open door on the driver’s side. Hereafter, the participant wasthen lifted up the LSB in a lateral position until the feet are released from under the steering column, allowing rotation onto back and then finally, slid into position further up the LSB (Fig. 1) **Self-extrication:** The casualty leaves the vehicle without assistance Study car preparation: The drivers door was opened Study vehicle: Seat Ibiza 5 door, 1999 Technique: The participant is asked to get out of the vehicle and take one step away. The fire crew offered no instructions on how the participant should exit the vehicle	

## Methods

This is an experimental crossover biomechanical study which builds on previous exploratory work and compares spinal movement at both the cervical spine and lumbar spine across each of four extrication techniques: (i) Roof removal extrication, (ii) B-post rip extrication, (iii) Rapid side door extrication, (iv) Self-extrication without instructions.

### Participants

Six healthy volunteers were recruited to participate in this study. The volunteers had no previous knowledge of extrication, had no back or neck conditions that may be exacerbated by extrication and had a mass of less than 100 kg. Participants were briefed on the study, had access to a participant information sheet in advance and completed written informed consent prior to participation.

### Data collection

Each participant’s height and weight were recorded prior to being fitted with the Inertial Measurement Unit (IMU) (Xsens Awinda; Xsens Technologies B.V., Enschede, Netherlands). The characteristics of IMU’s and their suitability to extrication research are described in our previous work [19]. The IMU sensor was attached to the head using a headband. The thorax was assumed to be rigid and sensors were positioned over the clavicular notch on the sternum, and over each scapula using a tight-fitting elastic vest. A sensor was positioned on the sacrum by attaching the sensor to shorts using hook-and-loop fastening, to prevent upward travel, and securing the sensor against the body with an elastic belt. Orientation data were collected from each sensor via a wi-fi link and sampled at a rate of 40 Hz. Collars were used throughout this study as we have previously demonstrated that they reduce movement during extrication [19]. The Laerdal (Laerdal Medical Corp., Stavanger, Norway) Stifneck collars were fitted by a member of the study team trained in their use in accordance with manufacturer guidance.

The vehicle type was pre-specified as a 5-door hatchback as this represents the commonest vehicle type on UK roads [20]. Three similar vehicles were used (Box 1). The same intact vehicle was used for the self-extrication and rapid side door extrication arms of the study, with separate pre-prepared vehicles being used for the side-rip and roof-removal arms of the study. Each of these vehicles were prepared with all extrication stages involving cutting equipment and removal of vehicle structure being completed before the study began (Box 1 and Fig. 1).

### Sample size

Previous work has identified self-extrication with collar and no instructions to be associated with the least spinal movement during self-extrication; we used the means and standard deviations to power this study [19]. Acknowledging its limitations, we used a minimally clinically important difference (MCID) derived from cadaveric work (2.7 mm) [21]. The power calculation was based on finding an anterior–posterior translational movement of 2.7 mm with a significance level of 1% and a power of 80%, giving a sample size per group of 57. At each stage, each extrication type was repeated a maximum of ten times with each of the 6 volunteers.

### Analysis

The IMU directly measures the segmental orientations from which relative motions can be calculated and reported, by assuming the relative rotations of adjacent vertebrae across the lumbar and cervical region are constant. Maximum excursions (movement from a hypothetical midline) were calculated for anterior/posterior (AP) and lateral (Lat) movement of the cervical and lumbar spine, respectively. In addition to reporting maximum excursions (the single largest movement) we report “travel”—the cumulative total of all movements throughout the extrication.

The time taken for extrication is also considered as a patient-orientated metric. Time for completion of each experiment was therefore also recorded, with the timer starting when the crew declared ready to begin and finishing when the patient was fully extricated and stationary.

Data were captured and analysed using the Biomechanics of Bodies (BoB Biomechanics Ltd,, Bromsgrove, UK) software interface before being exported to Excel (Microsoft v. 16.9) and SPSS (IBM v. 25, Armonk NY) for further analysis and reporting. Total excursions, standard deviation and confidence intervals are reported for each extrication type. P values were calculated using a two tailed t-test comparing each extrication method with the current standard (roof removal) extrication type.

The study protocol was reviewed and approved by the University of Coventry Research Ethics Committee (reference number P88416) and the University of Cape Town, Human Research Ethics Committee (reference number 530/2021).

## Results

Data from a total of 230 extrications were successfully collected for analysis (95.8% data capture success rate). Three of the six participants were female, with a mean age across all of the participants of 52 years (range 28–68) and BMI of 27.7 (range 21.5–34.6).

**Fig. 2:**
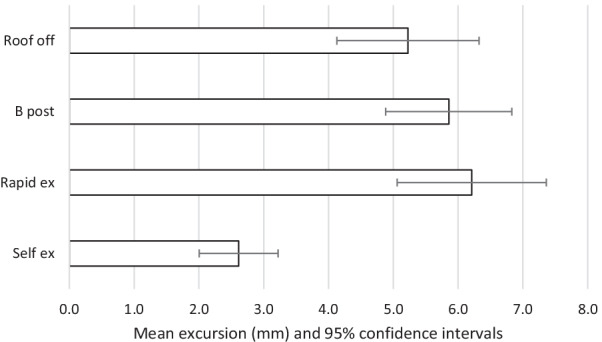
Mean excursion and confidence intervals for anterior–posterior movement at the cervical spine

**Fig. 3:**
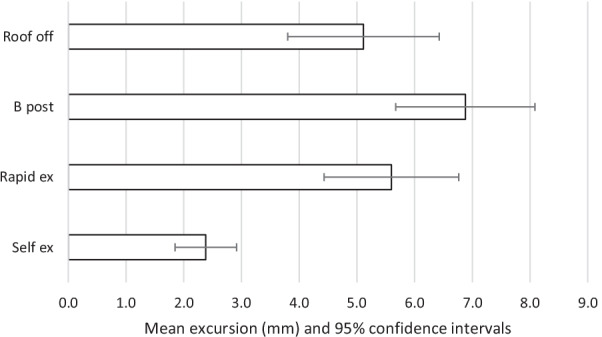
Mean excursion and confidence intervals for lateral movement at the cervical spine

**Fig. 4:**
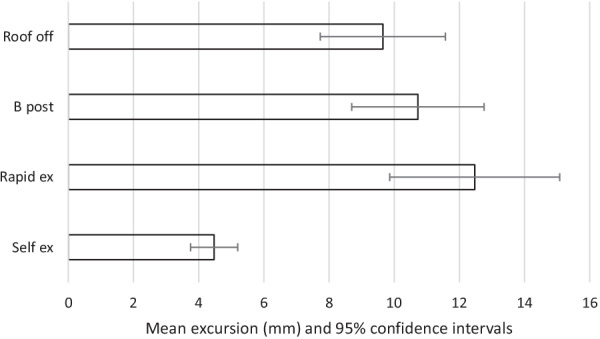
Mean excursion and confidence intervals for anterior–posterior movement at the lumbar spine

**Fig. 5:**
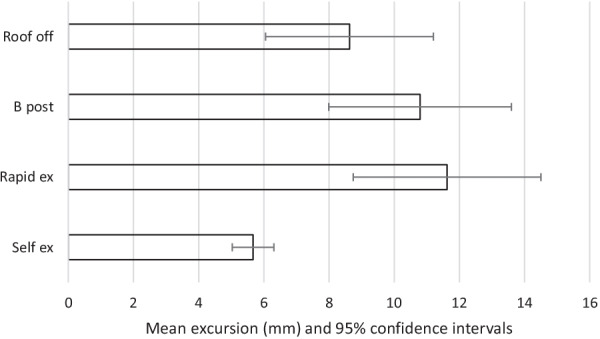
Mean excursion and confidence intervals for lateral movement at the lumbar spine

**Fig. 6:**
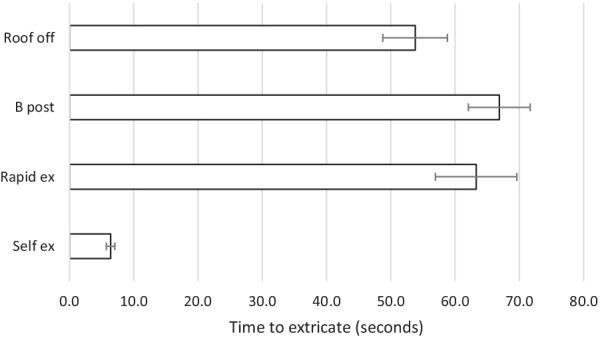
Time taken and confidence intervals (s)

The results are summarised in Tables 1, 2 and Figs. 2, 3, 4, 5, 6. The mean movements across the four extrication types were 4.4 mm (Cervical AP), 4.2 mm (Cervical Lat), 7.9 mm (Lumbar AP) and 7.8 mm (Lumbar Lat). Mean cervical roll was 16.6°, cervical pitch 12.4° and cervical yaw 17.1°. Mean lumbar roll was 16.6°, lumbar pitch 16.0° and lumbar yaw 25.4°.

**Table 1:** Participant demographics, extrications and mean AP movement

Participant	Sex	Age (years)	Weight (kg)	Height (cm)	BMI (kg/m^2^)	Extrications suitable for analysis	Mean AP cervical movement (mm)			
							Roof off	B post rip	Rapid	Self
1	F	40	89	167	31.9	39	4.2	7.0	11.0	2.2
2	F	52	100	170	34.6	38	7.6	7.8	6.5	6.9
3	M	57	89	168	31.5	39	6.6	4.8	7.8	3.0
4	F	28	62	167	22.2	36	7.4	3.9	6.7	0.9
5	M	68	80	181	24.4	38	2.5	5.1	2.3	1.2
6	M	57	69	179	21.5	40	3.0	6.4	3.1	1.6
		50.3	81.5	172.0	27.7	230	5.2	5.8	6.2	2.6

**Table 2:** Maximal movement and travel

	Maximal movement during extrication							Travel (total movement) during extrication						
	Roof off	B post	p value	Rapid	p value	Self	p value	Roof off	B post	*p* value	Rapid	*p* valve	Self	*p* value
Lumbar A/P[mm]	9.65	10.73	0.45	12.47	0.09	4.47	< 0.001	26.56	30.25	0.28	36.07	0.02	8.49	< 0.001
Lumbar Lat [mm]	8.63	10.79	0.27	11.62	0.13	5.67	0.03	21.80	30.70	0.06	37.67	0.008	10.69	< 0.001
Cervical A/P [mm]	5.23	5.86	< 0.001	6.21	< 0.001	2.61	< 0.001	16.69	17.72	0.65	20.51	0.13	4.97	< 0.001
Cervical Lat [mm]	5.11	6.88	0.05	5.60	0.59	2.38	< 0.001	14.56	19.02	0.09	17.68	0.28	4.46	< 0.001
Lumbar roll [°]	18.83	23.47	0.31	25.46	0.14	11.25	0.01	47.59	66.83	0.10	82.49	0.02	21.09	< 0.001
Lumbar pitch [°]	22.91	22.55	0.94	22.33	0.89	8.20	< 0.001	61.63	65.59	0.74	75.97	0.38	15.63	< 0.001
Lumbar yaw [°]	29.80	42.59	0.14	31.65	0.78	11.23	< 0.001	74.73	109.69	0.12	101.09	0.27	21.13	< 0.001
Cervical roll [°]	15.55	20.54	0.08	16.62	0.68	7.07	< 0.001	44.52	55.79	0.16	53.92	0.28	13.31	< 0.001
Cervical pitch [°]	14.90	16.29	0.48	17.55	0.21	7.34	< 0.001	47.32	48.67	0.82	56.51	0.15	13.99	< 0.001
Cervical yaw [°]	20.45	26.60	.098	22.98	0.53	6.10	< 0.001	52.46	69.31	0.07	64.41	0.25	12.14	< 0.001

For the cervical spine, the smallest overall movements were recorded during self-extrication (2.6 mm AP and 2.4 mm LAT). These were also the conditions producing the smallest movements at the lumbar spine (4.5 mm AP and 5.7 mm LAT).

The largest overall mean movements were seen in the cervical spine AP with the rapid side door extrication (6.2 mm). For cervical spine lateral movements, the side-rip resulted in the greatest movement (6.9 mm). For the lumbar spine, the greatest movement was recorded with the rapid side door extrication (12.5 mm AP and 11.6 mm LAT).

Self-extrication was significantly quicker than the other extrication methods (mean 6.4 s, Fig. 6). B-post rip extrication (66.9 s) was slower than roof-off (53.8 s) and self-extrication.

## Discussion

This is the first study to define spinal movements associated with each of the commonly used extrication techniques and to perform a powered comparative analysis. This study demonstrates that in healthy volunteers self-extrication results in significantly less movement at the cervical and lumbar spine than other extrication methods.

Results in relation to other studies: Biomechanical studies of extrication are widely heterogenous in design. Similar to the studies of Gabrieli and Dixon we find that self-extrication results in the smallest range of motion at the cervical spine – we offer additional data across a range of volunteers and movements [12, 13]. Dixon’s team also considered rapid extrication through the driver’s door and found as we did that this was associated with the largest movements of the techniques that they considered [12]. Ours is the first study to report movements with the ‘roof off’ technique or the B post rip which are commonly performed in the UK and in international practice [18].

Clinical and operational interpretation: Rescue service personnel are taught that unstable spinal injury should be assumed following an MVC and that traditional extrication techniques deliver minimal spinal movement, which are preferentially utilised because of this assumed benefit. As a result of this teaching, formal extrications are commonly performed for patients who could self-extricate [9].

This study demonstrates that self-extrication is associated with least spinal movement and the quickest time to extrication. Rapid, B-post rip and roof off extrication types are all associated with similar movements and time to extrication in preprepared vehicles.

Trapped patients are more likely to die than patients who are not trapped [8]. Trapped patients may have serious and time dependent injuries and therefore will benefit from an extrication technique which results in the minimum time spent in the vehicle [8]. Current operational practice favours techniques that are time consuming and do not result in the smallest possible patient movement—they do not achieve their intended objectives and as a result their use should be urgently reconsidered. In patients who can self-extricate, this should be the preferred method of extrication as it is associated with the smallest amount (maximal and total) of movement and least time. Self-extrication has many other secondary benefits including potential risk to patient and rescuer, human and equipment resource utilisation and minimises additional damage to the vehicles involved. An alternative extrication approach will be required for the very small minority of patients who are entangled in the vehicle or cannot self-extricate [8, 9]. Such patients are likely to be significantly injured and have time critical needs: for these patients, following disentanglement, the quickest deliverable extrication method should be chosen; the correct choice of technique in this context will depend on the actions required to disentangle the patient.

Strengths and weaknesses: Strengths of this study include efforts to maximise internal and external validity by recruiting male and female volunteers inexperienced in extrication with a range of weights, heights and ages. The study methods supported data collection from real vehicles, prepared as they would be for a ‘real life’ extrication, using active-duty rescue personnel. We successfully collected data from a large number of extrications to meet the pre-specified power calculation, supporting confidence in the reported results.

Our volunteers were uninjured, fully conscious and had not recently experienced a motor vehicle collision and did not have ‘true’ entrapment requiring disentanglement, as such the applicability of these results to the injured post collision population needs careful consideration. The volunteers were subjected to multiple extrications across a short time; we could find no evidence of ‘learning’ in the movements recorded but this could have influenced our results unknowingly. The rescue personnel also performed multiple extrications over the day—a far greater exposure than in operational practice. We did see faster extrications as the teams became increasingly familiar both with the techniques and working together as a team. Fatigue of the extrication team may also have influenced our results.

Further work: Additional biomechanical work could evaluate alternative extrication techniques (such as Scandinavian chain cabling [22]. Biomechanical models using healthy volunteers are unlikely to offer definitive answers; evolving technology has supported the collection of data in ‘near operational’ scenarios but is unlikely to be successful in collecting data on actual injured patients. As the paradigms of spinal immobilisation are challenged and additional data is made available as to the rarity of isolated unstable spinal injury in the context of other time critical injuries [8], those with responsibility for guidance and expertise in the area of extrication, trauma care and spinal injuries must work with patients and their representatives to evolve new approaches to extrication which improve the care of and outcome for our patients.

## Conclusions

In healthy volunteers, self-extrication is associated with the smallest patient spinal movement and the fastest time to complete extrication. Rapid, B-post rip and roof off extrication types are all associated with similar movements and time to extrication in preprepared vehicles. In patients who can self-extricate, this should be the preferred extrication method. In patients who can’t self-extricate, following disentanglement the most rapid method of extrication should be delivered.

## Data Availability

The datasets used and/or analysed during the current study are available from the corresponding author on reasonable request.
